# Mouse Mincle: Characterization as a Model for Human Mincle and Evolutionary Implications

**DOI:** 10.3390/molecules20046670

**Published:** 2015-04-15

**Authors:** Neela D. S. Rambaruth, Sabine A. F. Jégouzo, Hayley Marlor, Maureen E. Taylor, Kurt Drickamer

**Affiliations:** Department of Life Sciences, Imperial College, London SW7 2AZ, UK; E-Mails: n.rambaruth@imperial.ac.uk (N.D.S.R.); s.jegouzo@imperial.ac.uk (S.A.F.J.); hayleymarlor@hotmail.com (H.M.); maureen.taylor@imperial.ac.uk (M.E.T.)

**Keywords:** glycan-binding receptor, lectin, mycobacteria

## Abstract

Mincle, the macrophage-inducible C-type lectin also known as CLEC-4E, binds to the mycobacterial glycolipid trehalose dimycolate and initiates a signaling cascade by serving as a receptor for *Mycobacterium tuberculosis* and other pathogenic mycobacterial species. Studies of the biological functions of human mincle often rely on mouse models, based on the assumption that the biological properties of the mouse receptor mimic those of the human protein. Experimental support for this assumption has been obtained by expression of the carbohydrate-recognition domain of mouse mincle and characterization of its interaction with small molecule analogs of trehalose dimycolate. The results confirm that the ligand-binding properties of mouse mincle closely parallel those of the human receptor. These findings are consistent with the conservation of key amino acid residues that have been shown to form the ligand-binding site in human and cow mincle. Sequence alignment reveals that these residues are conserved in a wide range of mammalian species, suggesting that mincle has a conserved function in binding ligands that may include endogenous mammalian glycans or pathogen glycans in addition to trehalose dimycolate.

## 1. Introduction

After many years as an orphan receptor, the macrophage inducible C-type lectin, known as mincle or CLEC-4E, has recently been recognized as a receptor for glycans on pathogens [1,2,3]. It is of particular interest because it can bind to trehalose dimycolate, an unusual glycolipid in the outer wall of mycobacteria. Mincle is a prototype for a novel subgroup of animal glycan-binding receptors, because in addition to containing a Ca^2+^-dependent carbohydrate-recognition domain (C-type CRD) it initiates signaling in macrophages by interacting with the common Fc receptor γ subunit (FcRγ subunit) [4]. Binding of Syk kinase to the immunotyrosine activation motifs in the cytoplasmic domain of this accessory subunit results in activation of the CARD9 pathway, leading to secretion of inflammatory cytokines including interleukin 6 and tumor necrosis factor α [2,4,5].

A crystal structure of the CRD from cow mincle provides a structural basis for understanding the basis for binding of mincle to trehalose dimycolate [6]. One of the two glucose residues in the trehalose headgroup binds in a primary sugar-binding site that resembles that seen in other C-type CRDs, in which five amino acid side chains coordinate a Ca^2+^ and simultaneously four of these residues make hydrogen bonds with the 3- and 4-OH groups of the glucose, which is also ligated to the Ca^2+^. A secondary sugar-binding site interacts with the second glucose residue in trehalose and a hydrophobic groove next to the primary binding site is proposed to accommodate acyl chains attached to the 6-OH group of the glucose residue in the primary binding site. Although structural analysis of human mincle has suggested an alternative arrangement of the binding site [7], recent mutagenesis and binding studies indicate that the arrangement of the binding site in human mincle is likely to resemble closely that seen in the cow structure [8].

Important mycobacterial infections in humans include tuberculosis caused by *Mycobacterium tuberculosis* and leprosy caused by *M. leprae*, while *M. bovis* is the causative agent of the bovine form of tuberculosis [9]. Because of the medical and agricultural importance of these diseases and the potential role of mincle in mediating the interaction between macrophages and mycobacteria, the structure and mechanism of ligand binding by the cow and human proteins have been studied in detail. Although mycobacterial infection is not a common disease of mice, the experimental advantages of working with mice have made it a useful model system. An important advantage of working with mice is the availability of knock-out mice lacking mincle, FcRγ, and other components of the mincle signaling pathway [2,3,10]. In addition, macrophage cell lines from mice, such as RAW 264.7 cells, can be used for *in vitro* studies of mincle [11]. The applicability of the mouse model to understanding the role of mincle in human disease depends on the conservation of key properties between the human and murine proteins. The studies reported here were undertaken to investigate whether the biochemical properties of the human receptor are faithfully replicated by the mouse protein.

## 2. Results and Discussion

### 2.1. Expression of the CRD from Mouse Mincle

**Figure 1:**
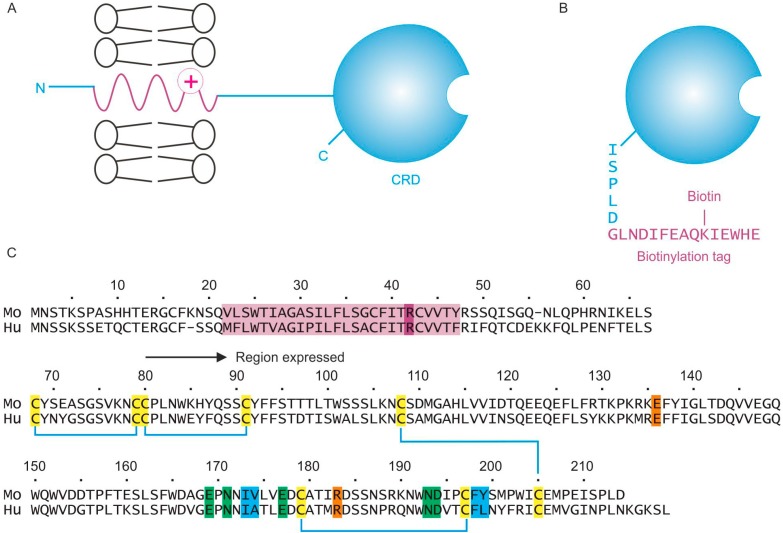
Organization of mouse mincle and expression strategy. (**A**), Summary of the native structure of mincle, containing a short *N*-terminal cytoplasmic domain, a transmembrane domain and a C-terminal CRD separated from the membrane by a short stalk; (**B**), The CRD was expressed in isolation, with a C-terminal biotinylation tag; (**C**) Comparison of the sequence of mouse and human mincle, showing the region of mouse mincle expressed in the current work, compared to the previously expressed human protein. The transmembrane domain is highlighted in purple, including a basic residue believed to be important for interaction with the FcRγ subunit. Six cysteine residues that form three disulfide bonds are highlighted in yellow, five amino acid residues that ligate Ca^2+^ and form the primary binding site for one residue of glucose in trehalose are in green, two residues that form a secondary binding site for the second glucose residue in trehalose are shown in magenta and four residues that are proposed to form a binding site for acyl groups attached to the 6-OH groups of trehalose are indicated in blue.

Mincle is a type II transmembrane protein, in which the CRD lies at the C-terminal end of the polypeptide (Figure 1A). The goal of these studies was to characterize binding to the CRD by expression of this region as a soluble protein separate from the rest of the polypeptide. In addition, appending a biotinylation tag to the C-terminal end of the polypeptide provides an efficient means of immobilization of the CRD to streptavidin-coated plates (Figure 1B). The region of the polypeptide to be expressed was selected based on previous studies of human mincle (Figure 1C). A cDNA encoding a minimal CRD, which is predicted to contain the three conserved disulfide bonds common to most C-type CRDs, was amplified from a full-length mouse mincle cDNA using primers that included the consensus biotinylation tag at the C-terminus.

**Figure 2:**
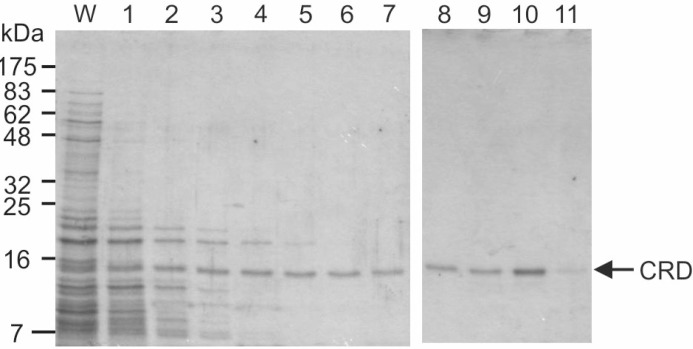
Purification of mincle CRD by affinity chromatography. Renatured mincle was applied to a 10-mL column of trehalose-Sepharose. Following washing with 10 mL of buffer containing Ca^2+^, the column was eluted with EDTA-containing buffer in 1-mL fractions. Aliquots (15 μL) of the final fraction collected during the wash (W) along with the elution fractions were analyzed by SDS-polyacrylamide gel electrophoresis. Protein was visualized by Coomassie blue staining. The expected molecular weight of the CRD with the biotinylation tag is 17.8 kDa.

The cDNA was inserted into a vector containing the T7 promoter and co-expressed with the biotin ligase gene to ensure efficient attachment of biotin to the lysine residue in the C-terminal tag. Correct folding of C-type CRDs requires both formation of the network of disulfide bonds and binding of Ca^2+^. Some C-type CRDs, including the CRD from human mincle, have been expressed directly as folded proteins after being directed into the periplasm of bacteria by a prokaryotic signal sequence [12]. In the case of mouse mincle, this strategy did not produce protein in sufficient yield for functional studies, so an alternative approach was employed in which the protein was expressed as inclusion bodies in the cytoplasm of *Escherichia coli*. Following solubilization of the denatured protein in guanidine hydrochloride, it was refolded by dialysis against Ca^2+^-containing buffer. The correctly folded protein was selectively purified by affinity chromatography on columns of trehalose-Sepharose, prepared by divinyl sulfone coupling to achieve a high density of immobilized sugar (Figure 2). The presence of some protein in the early fractions suggests that it does not bind tightly to the resin, although a significant amount of the protein is retained sufficiently that it elutes in fraction 10, as the EDTA-containing buffer emerges from the column.

### 2.2. Binding of Mouse Mincle to Sugar Ligands

A solid phase binding competition assay for quantification of the affinity of mouse mincle for sugar-containing ligand was developed, based on immobilization of the CRD on streptavidin-coated wells. Radioiodinated Man-bovine serum albumin (BSA) was used as a reporter ligand. As shown below, simple monosaccharides are not high affinity ligands for the CRD, binding of Man-BSA can be detected because at a sugar density of approximately 31 mannose residues per protein molecule, the Man-BSA is able to bridge between multiple CRDs immobilized in clusters on the tetrameric streptavidin. When working at concentrations of Man-BSA well below saturation binding, inhibition constants obtained in this assay will correlate closely with dissociation constants [13].

**Figure 3:**
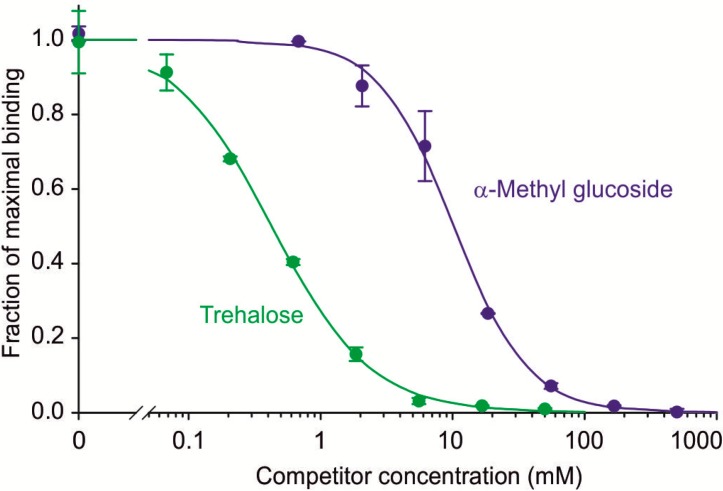
Competition binding assays comparing binding of simple sugars to mouse mincle. Biotin-tagged mincle immobilized in streptavidin-coated wells was probed with ^125^I-Man-BSA in the presence of various concentrations of competing ligands. The data were fitted to a simple binding equation [14] using a non-linear least squares fitting program to determine K_I_ values. Circles represent experimental values, with standard deviations for individual data points indicated by error bars, and the fitted curve is shown by the solid line.

Competition assays comparing α-methyl glucoside and trehalose yielded K_I_ values of 10.3 ± 1.0 mM and 0.54 ± 0.18 mM (Figure 3), indicating that mincle binds trehalose with roughly 19-fold higher affinity than it binds glucose. This result is very similar to the ratio of 17 obtained for the affinities of human mincle for trehalose and α-methyl glucoside [8] although the absolute affinity of mouse mincle for trehalose is somewhat higher than for human mincle (K_I_ value of 1.34 ± 0.18 mM). This finding suggests the presence of primary and secondary sugar-binding sites in mouse mincle that closely resemble those in the human protein, an interpretation that is consistent with conservation of the residues that form both of these sites (Figure 1C).

**Figure 4:**
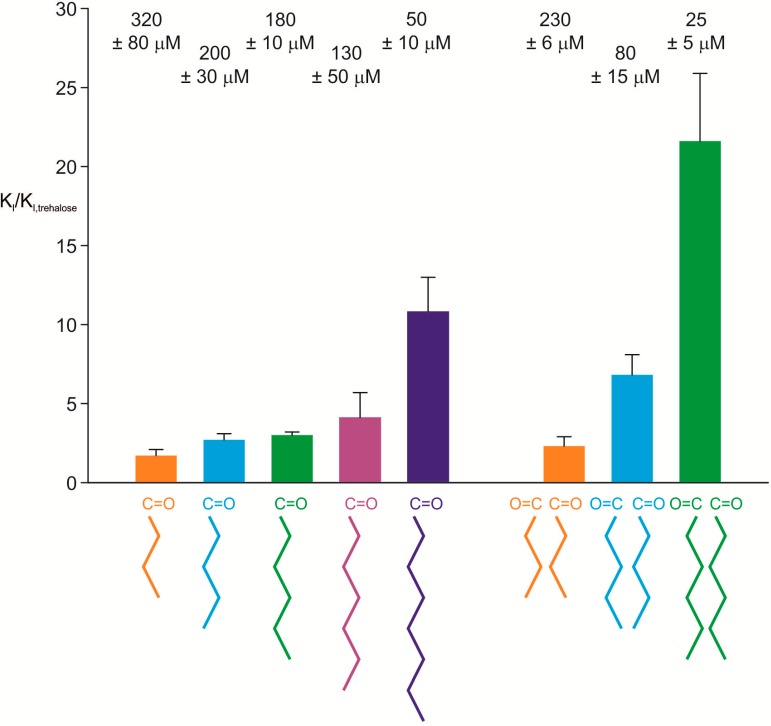
Summary of results for mouse mincle binding to mono- and di-acyl derivatives of trehalose. Affinities for trehalose derivatives were determined using the competition binding assay. The results are expressed as means ± standard deviations, both numerically as K_I_ values at the top and graphically as relative affinities compared to trehalose at the bottom.

In a further comparison with the human form of mincle, the mouse protein was tested for binding to a series of acylated derivatives of trehalose that serve as simplified, soluble analogs of the natural trehalose dimycolate ligand. These compounds, in which one or both of the 6-OH groups are derivatized with short chain fatty acids, were generated using the ability of the lipase from *Candida antarctica* to attach short chain acids selectively to primary alcohol groups under anhydrous conditions [15]. Comparison of the K_I_ values for mono-acyl derivatives shows increasing affinity with increasing chain length up to 10-fold higher affinity than simple trehalose (Figure 4). A similar trend is observed for the di-acyl derivatives and these show significantly enhanced affinity compared to the mono-acyl derivatives. This trend, which is both qualitatively and quantitatively similar to that previously seen for the human protein [8], has been attributed to the presence of a hydrophobic groove adjacent to the 6-OH group of the glucose in the primary binding site in the mincle CRD. The hydrophobic character of the four aliphatic and aromatic residues that form this groove is conserved in the human and mouse proteins (Figure 1C), so the shared ability to bind the glycolipid analogs is consistent with the presence of the groove in both proteins.

**Figure 5:**
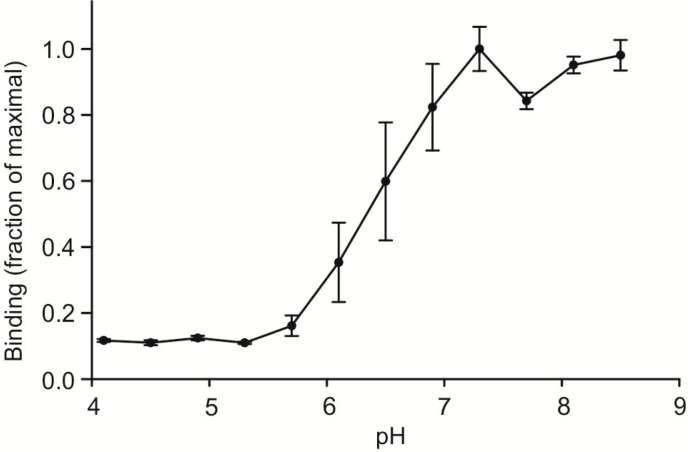
pH dependence of ligand binding to mouse mincle. Binding of ^125^I-Man-BSA to immobilized CRD from mouse mincle was determined in a series of pH buffers formed with a mixture of 25 mM MES and 25 mM MOPS. Data are presented as mean values ± standard deviations. A midpoint of approximately pH 6.5 was obtained with two independently prepared sets of buffers.

A final comparison of the characteristics of human and mouse mincle was made by testing the effect of pH on binding. Binding is maximal above pH 7 and falls of in the range between pH 6 and 7, so that is it reduced to 10% of maximal binding at the pH of intracellular endocytic compartments such as endosomes (Figure 5). Although it has been suggested that mincle probably lacks endocytic activity [2], the pH profile is very similar to that seen for other glycan-binding receptors that recycle through endosomes [16] and it is interesting that the property is retained across species.

### 2.3. Comparison of Mincle across Species

**Figure 6:**
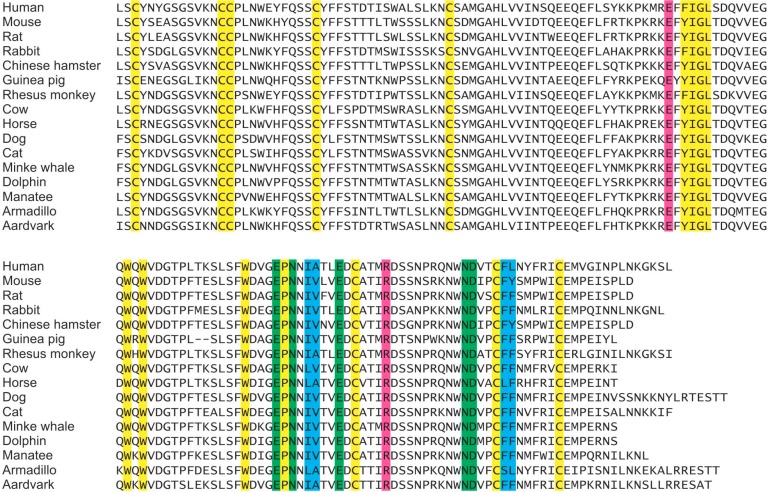
Sequence alignment and conservation of key residues in the CRD of mincle across species. A selection of the 62 sequences of mincle orthologs spanning a range of species is shown. Some of the key conserved framework residues common to C-type CRDs are highlighted in yellow, while the residues involved in binding are indicated as green at the primary binding site for the first residue of glucose in trehalose, magenta at the secondary binding site for the second glucose residue in trehalose and blue at the hydrophobic groove that is proposed to form a binding site for acyl groups attached to the 6-OH groups of trehalose.

Based on the combination of previous structural work on the cow protein and mutagenesis of the cow and human proteins, the binding properties have been mapped to specific residues in the CRDs of these proteins, and the present study confirms that mouse mincle, in which these same residues are present, shows conserved binding properties. The strong similarity in the ligand-binding properties of mouse and human mincle suggested that these characteristics might be conserved over a broader range of species. A wider survey of mincle conservation was undertaken, comparing all entries in the National Center for Biotechnology Information database. A total of 62 different sequences that appear to be orthologs of mincle were obtained. Alignment of a selection of these sequences is shown in Figure 6.

In all 62 sequences, there is complete conservation of the residues that form the primary and secondary binding sites as well as conservation of the hydrophobic character of the side chains that make up the hydrophobic groove in cow mincle. Based on the analysis of mouse mincle presented here, in combination with previous work on the human and cow proteins, these findings suggest that the binding properties of mincle are likely to be conserved across these species.

### 2.4. Discussion

Two key findings emerge from this work regarding comparison of the human and mouse proteins and conservation of mincle across species. The results provide validation of mouse as a model system for studying mincle function in binding trehalose dimycolate. This situation contrasts with the notable lack of conservation of ligand-binding properties in some pathogen-binding receptors, such as DC-SIGN (dendritic cell-intracellular adhesion molecule 1-grabbing nonintegrin), which is one of two closely related receptors in humans, compared to a family of eight closely related genes in mouse, none of which encodes a direct ortholog of the human proteins [17]. From an experimental standpoint, this result provides a basis for extrapolating results with trehalose dimycolate and its analogs obtained in mice, such as the effects of knocking out the mouse gene for mincle, to understanding human biology. Of course, the responses mediated by mincle may still differ as a result of other differences in the immune system between species.

Recent studies of mouse and human mincle in macrophages and in transfected cells indicate that their interactions with some other types of ligands, such as glycerol monomycolate, differ [18]. In these experiments, receptors on cells were exposed to surfaces coated with the ligand, so in addition to possible differences in affinity of the CRDs for these chemically distinct ligands, the differences observed may also be influenced by differences in cell surface density or disposition and interactions with other molecules in the plasma membrane. Nevertheless, the results suggest that biochemical characterization of the interaction of additional types of ligands with the CRDs from human and mouse mincle will be informative.

The demonstration that conserved sequences correlate with conserved functional properties provide a basis for speculation about evolution of mincle function. One important point about the mincle sequences that are available is that mincle orthologs are readily identified across mammalian species, but not beyond this in birds, reptiles or fish. Such selective radiation of some glycan-binding receptors within groups of species has been seen at other levels, such as receptors found in vertebrates but not in invertebrates [19]. The results for mincle suggest that it emerged as a mammalian-specific feature of the adaptive immune response. A further point about mincle evolution relates to the selective pressures that are proposed to drive divergence of the sugar-binding receptors. It has been proposed that rapid divergence of receptors such as DC-SIGN and its homologs in different species has resulted from exposure of these species to different sets of viruses, bacteria and fungi, so that the receptors are adapted in evolutionary time to cope with different pathogens [20,21]. Conservation of ligand-binding properties, as suggested by the work reported here, is more commonly associated with receptors that bind endogenous mammalian glycans, which would not be subject to such evolutionary pressures.

There are several potential explanations for the seemingly unusual evolutionary history of the CRD in mincle. One possibility is that the mycobacterial threat has been persistent across species, so the properties of mincle have been retained. Mycobacterial infections have been reported in a very wide range of species, but for the most part these appear to be a result of infection from humans or other natural hosts and are not self-sustaining infections except in a few species, such as humans and cows [22,23,24]. It is also notable that such infections have been observed in birds and fish, in which the genomes do not seem to encode mincle orthologs. Another possibility is that the conservation of mincle reflects binding to other ligands, on widely distributed pathogens or on endogenous glycoproteins or glycolipids, and that the binding of mincle represents an opportunistic exploitation of this conserved property of mincle by mycobacterial species. The absence of high affinity binding of both human and mouse mincle to panels of mammalian glycans present on the glycan array created by the Consortium for Functional Glycomics [25] is more consistent with the idea that mincle targets primarily pathogen glycans, but there is also evidence that mincle binds non-glycosylated endogenous ligands such as spliceosome-associated protein 130 released from necrotic cells [4].

## 3. Experimental Section

### 3.1. Materials

Sources of material for molecular biology were as follows: a clone of mouse mincle generated by the Integrated Molecular Analysis of Genomes and their Expression (IMAGE) Consortium, Clone 3158063/IRAVp968D122D, with the sequence deposited under accession number BC003218 in the National Center for Biotechnology Information database, was obtained from Source BioScience; synthetic primers, pCR2.1-TOPO cloning vector from Life Technologies (Glasgow, UK); restriction enzymes from New England Biolabs (Hitchin, UK). Trehalose-Sepharose resin for affinity chromatography was prepared by divinyl sulfone coupling [26]. Streptavidin-coated polystyrene wells for the binding assay were from Thermo Scientific Pierce (Loughborough, UK). Man-BSA, obtained from E-Y Laboratories (San Mateo, CA, USA), was iodinated by the chloramine T method (Greenwood and Hunter [27]). Synthesis and characterization of acylated trehalose derivatives has been previously documented [6]. All of these compounds are soluble in water at levels at least 10-fold higher than the concentrations used in the assays.

### 3.2. Cloning of cDNA for Mouse Mincle

The cDNA for expression of mouse mincle was amplified with Advantage 2 polymerase mix (Takara Bio, Saint-Germain-en-Laye, France) from the IMAGE clone using forward primer aaggatccgatcttggaggatgattaaatggcttgtcctttgaactggaaacattatc, encoding a linker for the expression vector, and reverse primer taaagcttctactcatgccactcgattttctgtgcttcgaagatgtcattcagtccgtccagaggacttatttctggc, encoding the biotinylation tag. The resulting cDNA was purified by agarose gel electrophoresis and cloned into the vector pCR2.1-TOPO (Life Technologies, Glasgow, UK). The sequence was confirmed on an Applied Biosystems 310 genetic analyzer (Foster City, CA, USA). The *BamH*1-*Eco*R1 insert was excised and inserted into the vector pT5T [12,28] for expression under the control of phage T7 RNA polymerase.

### 3.3. Expression of Biotin-Tagged CRD

The expression vector and plasmid pBirA, which encodes biotin ligase and the chloramphenicol resistance gene [29], were transformed into *E. coli* strain BL21(DE3) and were grown in Luria-Bertani broth in the presence of 50 μg/mL ampicillin and 20 μg/mL chloramphenicol. A 200-mL starter culture grown at 30 °C overnight was used to inoculate 6 L of medium. Growth was continued at 37 °C with shaking until an A_550_ of 0.7 was reached. Isopropyl-β-D-thiogalactoside was added to a concentration of 100 mg/liter followed by growth for a further 150 min at 37 °C. Bacteria were harvested by centrifuging at 4000× *g* for 15 min at 4 °C and were washed by resuspending in 10 mM Tris-HCl, pH 7.8 and re-centrifuging and the resulting bacterial pellets were stored frozen.

### 3.4. Purification of Biotin-Tagged CRD

Frozen bacterial pellets were thawed, suspended in 30 mL of 10 mM Tris-HCl, pH 7.8, and lysed by sonication for four times at full power on a model 250 sonicator (Branson, Danbury, CT, USA) with cooling on ice after each 1-min treatment. The insoluble protein was collected by centrifugation at 10,000× *g* for 15 min at 4 °C and solubilized by homogenization in 100 mL of 6 M guanidine-HCl containing 100 mM Tris-Cl, pH 7.0 and incubated in the presence of 0.01% 2-mercaptoethanol for 30 min at 4 °C. Following centrifugation for 30 min at 100,000× *g*, protein was renatured by dialysis against 3 changes of 0.5 M NaCl, 25 mM Tris-Cl, pH 7.8, 25 mM CaCl_2_ at 4 °C. Insoluble material was removed by centrifugation for 30 min at 30,000× *g*. The final supernatant was applied to a 10-mL column of trehalose-Sepharose. The column was rinsed with 10 mL of 150 mM NaCl, 25 mM Tris-Cl, pH 7.8, 25 mM CaCl_2_, the bound protein was eluted with 10 mL of 150 mM NaCl, 25 mM Tris-Cl, pH 7.8, 2.5 mM EDTA. Fractions of 1-mL were collected and aliquots were examined by SDS-polyacrylamide gel electrophoresis to identify those containing the CRD.

### 3.5. Analytical Methods

SDS-polyacrylamide gel electrophoresis on 17.5% polyacrylamide gels was performed by the method of Laemmli [30]. Streptavidin coated plates were washed once with binding buffer (150 mM NaCl, 25 mM Tris-Cl, pH 7.8, 2.5 mM CaCl_2_) and were coated with biotin-tagged CRDs at 5 μg/mL in 0.1% (*w*/*v*) BSA in binding buffer for 2 h at 4 °C. Wells were washed three times with binding buffer before addition of ligands. Serial 3-fold dilutions of competing ligands were prepared in water, after which buffer and reporter ligand were added to achieve final concentrations of 0.5 μg/mL ^125^I-Man-BSA and 0.1% BSA in binding buffer. This concentration ^125^I-Man-BSA is at least 100-fold lower than its K_D_, ensuring that the K_I_ values obtained accurately reflect the affinities of the competing ligands independent of the concentration of the reporter ligand [13]. In the pH-dependence assays, buffers containing 25 mM 2-(*N*-morpholino)ethanesulfonic acid and 25 mM 3-(*N*-morpholino)propanesulfonic acid were used in place of the Tris buffer. After incubation for 2 h at 4 °C, wells were washed three times with binding buffer and bound radioactivity was measured in a Wallac WIZARD gamma counter (PerkinElmer Life Sciences, Beaconsfield, UK). Inhibition constants were obtained using SigmaPlot (Systat Software, Hounslow, UK). Values reported are means ± S.D. for 2–3 replicate experiments.

### 3.6. Sequence Comparisons

Potential mincle orthologs were identified in the protein section of the National Center for Biotechnology Information database [31] by searching with the terms mincle, CLEC4E, and CLECSF9. 

## 4. Conclusions

The results presented here provide evidence that key properties of mincle are conserved between mice and humans. Specifically, the interaction of the CRD with acylated trehalose analogs of trehalose dimycolate shows similar specificity and affinity in the binding site. The results are consistent with the conservation of amino acid residues that form the ligand-binding site in human mincle as well as the cow ortholog. The fact that these residues are conserved across mammalian species suggests that mincle has a conserved function in binding this class of ligands.
